# Sedation of Adults with Orally Administered Midazolam in Dentistry – A Retrospective Study

**DOI:** 10.2340/aos.v83.41403

**Published:** 2024-09-19

**Authors:** Marika Storskrubb, Pia Gabre

**Affiliations:** a Department of Plastic & Oral and Maxillofacial Surgery, Department of Surgical Sciences, Uppsala University, Uppsala, Sweden; b Department of Orofacial Medicine, Public Dental Health, Region Uppsala, Sweden

**Keywords:** Adults, dental care, midazolam, retrospective study

## Abstract

**Objective:**

The use of midazolam (MZ) has increased in dentistry, but the effect in adults is sparsely studied. The aim of this study was to investigate doses, effects, and side effects of orally administered MZ as a sedative for adults in a dental care organization.

**Material and methods:**

A retrospective record review was performed including all adult patients in the dental care organization ≥20 years receiving MZ, identified through a logbook for addictive drugs, during 2020. From patients’ records, the following data were extracted: age, gender, medical history, reason for sedation, performed treatments, doses, effects, and side effects of MZ.

**Results:**

In total, 265 patients on 418 occasions had been sedated, which constituted 2.3 sedations per 1,000 visits and 3.3 sedations per 1,000 treated patients. Mean age was 40.8 years and 67.7% were females. The most common reason for sedation was anxiety. Mean dose in primary dental clinics was 7.9 mg and in specialist clinics, 6.8 mg (*p* < 0.001). Older patients (>70 years) had lower doses than younger (*p* < 0.001), while no difference was found between ages 55–70 years and those who were younger. Dental treatment was completed in 91.9% of occasions, and side effects were registered in 2.2%. Successful dental treatment was related to type of treatment performed.

**Conclusions:**

Sedation is rarely used, particularly in primary dental care, and the use varies widely between clinics. MZ administered by dentists seems to be safe and effective. A sedation record should be used to make patient data such as weight and medical conditions available.

## Introduction

Occasionally in dental care, patients need sedation before or during examinations and treatments. This may apply to patients who have difficulty cooperating in dental care due to low age, disabilities, or cognitive and intellectual impairments [1, 2]. Also, patients with severe or moderate dental anxiety, reported to be 4.7 and 4.5%, respectively, in a population, could be supported by sedation [3]. Although the prevalence of anxiety seems to have decreased in the population during the last five decades, the proportion of individuals who report severe anxiety has remained on the same level [3]. Anxiety in patients can sometimes be reduced by behavioral interventions [4, 5], but in some cases, pharmacological treatments will still be required [6]. Two Canadian studies has reported that dentists underestimate patients’ preference for sedation [7] and that dentists express several barriers for offering sedation to their patients [8].

When midazolam (MZ) is administered orally, the aim is mainly to achieve a moderate level of sedation. In accordance with practical guidelines from several associations published in the journal Anesthesiology 2018, moderate sedation is defined as ‘a drug-induced depression of consciousness during which patients respond purposefully to verbal commands, either alone or accompanied by light tactile stimulation’ [9]. Spontaneous ventilation is adequate, and therefore no measures for respiratory support need to be taken in most cases. MZ belongs to a group of benzodiazepines used to induce sedation, a state of calm, drowsiness, or sleep and reduces anxiety and muscle tension. It has antispasmodic effects and can cause some memory loss [10]. Since the 1990s, the use of MZ has increased in dentistry and has largely replaced the use of diazepam [11]. MZ can be administered intravenously, intramuscularly, rectally, intra-nasally, or orally [12]. The latter is the most common method of administration in dental care. The advantages of MZ are, compare to other benzodiazepines, a short half-life of 1–2 h, short on-set time, and administration as an oral solution [10].

The effect of MZ in dentistry is examined in several studies, mainly in children and adolescents. However, Rignell et al. [2] reported that orally administered MZ was effective and safe in dental treatment of people, with an average age of 80 years, with major neurocognitive disorders. In 2016, a systematic review based on 30 studies investigating the effectiveness of MZ administered before diagnostic and therapeutic medical procedures, reported inconsistent evidence that oral MZ decreased anxiety compared to placebo [13]. Five years later, the review was updated, and another eight studies could be identified [11]. Although quality of evidence was not considered to be high, the conclusion was that oral MZ reduced the risk of difficulties in performing dental procedures compared to placebo. In addition, MZ reduced discomfort/pain compared to placebo.

Before sedation patients’ state of health should be assessed using the ASA classification [14]. Several studies state that sedation with oral MZ is safe to use in patients with an ASA classification 1–2 [12, 15, 16]. Sedation with benzodiazepines results in muscle relaxation and reduced respiration [10]. Several conditions are therefore seen as contraindications to sedation with MZ, absolute or relative, such as sleep apnea syndrome and severe lung disease [9].

With correct dose in healthy patients or those with less serious diseases, the risk of serious sedation complications is very low [2, 12, 15]. During MZ sedation, the complication paradoxical reactions, for example, causing patients to become hyperactive or aggressive, can occur in 2–12% of treatments [15, 16].

The aim of this observational study was to investigate the use of orally administered MZ as a sedative for adults in a dental care organization in a retrospective record review. We investigated patient characteristics, such as age and general condition as well as the type of dental procedure performed, MZ doses used, achieved sedative effects, and side effects. The analytical part of this study aimed to assess the impact of patient and procedural characteristics on the risk of not being able to complete the planned dental treatment.

## Materials and methods

To fulfill the aim, we performed a retrospective record review study including all adult patients who received orally administered MZ during 2020 in the Public Dental Health in Region Uppsala, Sweden. There are 23 public dental care clinics in Uppsala, three of which are for specialist dental care for adults and 20 primary dental clinics. The focus of the specialist clinics is orofacial medicine (two clinics) and a clinic for prosthodontics, endodontics, periodontology, and temporomandibular disorders. A specialist clinic for maxillofacial surgery, located at the university hospital, does not organizationally belong to Public Dental Health in Region Uppsala and is therefore not included in the study. The study was approved by the Swedish Ethical Review Authority (Dr 2021-03274) as a registry study, which means that the record review could be done without requesting informed consent from the individual patient.

### Clinical procedures and guidelines

As there are no national guidelines for sedation in dentistry, Public Dental Health in Region Uppsala, like other regions in Sweden, has developed own guidelines. The guidelines describe indications, contraindications, and clinical procedures to support clinicians when sedating patients [17]. It also contains a dose chart to adapt doses to age and weight: as drug metabolism is impaired due to reduced renal and hepatic clearance with increased age, the dosage requires age adjustment [18]. For clinicians to document important facts such as weight, time of administration, dose, and saturation during treatment, the guidelines provide a separate sedation template to be inserted into patients’ records.

Most clinics use sedation with MZ when patients receive regular dental care or care in emergency events for patients with poor cooperation and/or anxiety. In specialist dental clinics the participants had been referred to the clinic or, in clinics specializing in orofacial medicine, the participants received their regular dental care at the specialist clinic. All dentists who performed treatments with MZ had received 1 day of education from an anesthesiologist. Dentist specializing in orofacial medicine had a further 2-weeks’ training in using MZ and nitrous oxide. In addition, all staff were regularly trained in cardiopulmonary resuscitation. Pulse oximeters for measuring oxygen saturation and equipment to provide oxygen via mask when needed were available in all clinics. Dentist calculated the dose based on the guidelines and administered the liquid to the patients, sometimes with help of a syringe back in the mouth or mixed in lemonade. The guidelines recommend waiting at least 20 min before the treatment commenced. The clinics register all use of addictive drugs administered to patients in a special logbook, which is regularly followed up.

### Data collection

All clinics were asked to send a copy of the logbook for addictive drugs from the year 2020 to the researcher responsible for the study. From this logbook, patients who could be included in the study were identified. Inclusion criteria were as follows: individuals born 1st January 2000 or earlier who received MZ one or more times during the year 2020 for whom the treatment was registered in a logbook for addictive drugs at one of the Public Dental Health clinics in Region Uppsala.

Exclusion criteria were individuals with restricted medical records or who could not be identified based on the information in the logbook. Another reason for exclusion was that, although drug withdrawal was registered in the logbook, there was no note in the medical record that the patient had received MZ. All individuals identified through the drug administrative logbook according to the criteria above constitute the study population. If the clinic had not sent copies of the drug logbook according to instructions, or in cases where the logbook was missing at the clinic, the clinic’s patients were excluded from the study.

Data collection from medical records was undertaken for each sedation occasion. The following information was registered in an examination protocol developed for the study:

Age and gender;

Medical history and ASA classification;

Reason for sedation;

Performed treatment;

Dose and effect of MZ;

Weight;

Side effects and measures in case of side effects;

Postoperative recovery;

Other data noted about the sedation in the medical record such as fasting routines, duration of treatment, vital parameters, contraindications, etc.

### Handling of data and statistical processing

The research started with a pilot study consisting of 20 medical records where the protocol was tested based on which data were possible to collect in the medical records. Use of separate sedation template facilitated the collection of structured data. To be able to handle narrative text in the medical records in a structured way, appropriate categories were created for several variables based on the results in the 20 test protocols, for instance effect of sedation and side effects. Data that could not be found in the records were categorized as ‘not described’. The categories are shown in tables and figures in the result section.

The examination protocols were provided with codes that could not be traced to any specific patients before data were transferred in a de-identified format to a database. Agreement between the protocols and the database was tested for 42 (10%) randomly selected subjects (Random Number Generator). This test of the quality of the transferring process showed a disagreement of 0.045%. The data were compiled and reported descriptively. Statistical analyses were performed to evaluate differences concerning gender, ages, and doses of MZ. In addition, the relationship between the effects of the sedation and possible side effects was tested. For continuous variables, t-test was used for comparison between two groups (age and doses). Linear regression analysis was used to investigate a potential difference in 1) age between female and male patients, 2) administrated doses between specialist care and primary dental clinics, and 3) administrated doses between age groups. We used clustered robust standard errors to correct for dependence between visits within each patient. To assess potential differences between patients who completed the planned dental treatment and those who did not, we used the two-sample t-test and Fisher’s exact test. These analyses were based on the first visit, during the study period, for each patient. The level of significance was 0.05.

## Results

Of the 23 dental clinics in Public Dental Health in Region Uppsala in Sweden, 22 clinics could deliver sedation data from the year 2020, which means that 94.8% of all visits in the region could be included in the study. In total, 265 adult patients had been sedated on 418 occasions. During the same period, 179,784 visits had taken place at the 22 dental clinics and 80,740 unique adult patients, that is, each individual counted only once during the studied period, had received care. This means that 2.3 sedation sessions were performed per 1,000 visits and 3.3 sedations per 1,000 treated patients (Table 1). As described above, one clinic could not deliver sedation data, and the reason was that no logbook could be submitted. Two more clinics did not contribute sedation data because no adults had been sedated at the clinic in 2020. No records were excluded due to restricted medical records or that the patient could not be identified in the logbook. In 16 patients, there was no note in the medical record of having received MZ although drug withdrawal was registered in the logbook. The remaining 20 clinics were of varying size and had the number of patient visits ranging from 2,264 to 15,407 and the number of unique patients from 745 to 7,418. The number of sedations per 1,000 visits and unique treated patients ranged from 0.30 to 24.29 and 0.66 to 46.98, respectively. Specialist clinics conducted sedations more frequently (Table 1). Most patients had only one sedation session during the year while one-third (32.5%) had more than one.

**Table 1:** Description of sedation treatments data collected retrospectively from medical records.

	Variable	Number
**Total number of sedations:**		
	Primary dental care, per 1,000 visits (range)	1.30 (0.30–3.93)
	Special dental care, per 1,000 visits (range)	9.89 (5.74–24.29)
	Primary dental care, per 1,000 unique patients (range)	2.03 (0.66–4.03)
	Special dental care, per 1,000 unique patients (range)	15.6 (9.59–46.98)
**Gender:**		
	Females (%)	283 (67.7)
	Males (%)	135 (32.3)
**Age:**		
	Mean total, years (range)	40.8 (20–84)
	Mean primary care, years (range)	38.2 (20–71)
	Mean specialist care, years (range)	43.1 (20–84)
**ASA-classification:**		
	ASA class 1 (%)	238 (57.0)
	ASA class 2 (%)	58 (13.8)
	ASA class 3 (%)	4 (1.0)
	ASA class 4 (%)	0 (0)
	ASA not described (%)	118 (28.2)
**Reason for sedation:**		
	Dental fear (%)	216 (51.7)
	Difficult treatment (%)	2 (0.5)
	Disability (%)	59 (14.1)
	Feeling nauseous (%)	10 (2.4)
	Patient’s request (%)	14 (3.3)
	Not described (%)	117 (28.0)
**Treatment:**		
	Tooth extraction (%)	83 (19.9)
	Surgical operation (%)	82 (19.6)
	Filling (%)	101 (24.1)
	Depuration (%)	37 (8.9)
	Root canal treatment (%)	78 (18.7)
	Emergency visit (%)	20 (4.8)
	Others (%)	13 (3.1)
	Not described (%)	4 (0.9)

Sedation occasions *n* = 418.

**Figure 1:**
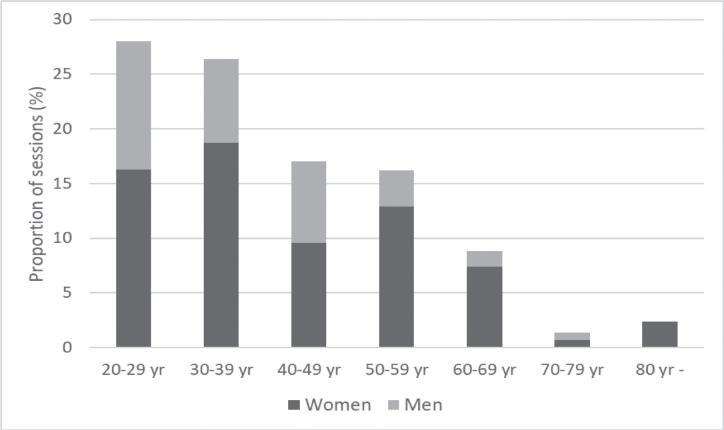
The distribution in age groups related to gender (%). Sedation occasions *n* = 418. Linear regression analysis, *p* = 0.038.

Females received sedation more often than males during the year of study. Mean age was 40.8 years with a range of 20–84 years (Table 1). More than half of the sedations (54.3%) were given to people under the age of 40 years (Figure 1). Female patients (mean age 42.4 years) were older than male patients (mean age 37.2 years) (*p* = 0.038). The main reason for giving sedation was dental fear (51.7%) followed by disability (14.1%). In only two sedations, severe treatments were given as a reason, and in none, the medical risk as reason was mentioned. The reason for sedation was not described in more than a quarter of occasions (Table 1). When the reason for sedation was disability, 54.2% of the sedated patients were males compared with 32.2% males in the total sample. The indication disability also differed for certain treatments, for example, patients with disability constituted 71.0% (data not shown) of those who received depuration compared to14.1% for the whole sample (Table 1). Restorative treatments such as fillings and crowns were the most common treatments performed during sedation. Also tooth extractions, surgical operations, and root canal treatments occurred frequently in conjunction with sedation (Table 1). If several different treatments were performed during the sedation, only one treatment was registered. In those cases, the treatment assessed with the highest degree of severity was recorded in the following order: surgical operations, tooth extractions, root canal treatment, fillings, depuration, then emergency visits.

**Figure 2:**
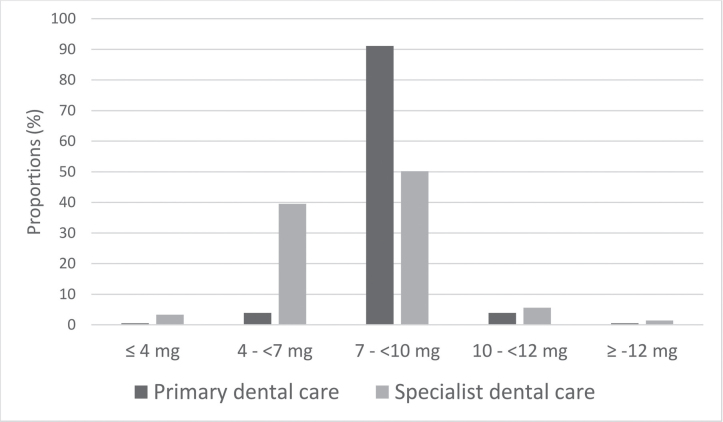
Percentage distribution of given doses of MZ in primary dental care and specialist dental care. Sedation occasions *n* = 418. Linear regression analysis, *p* < 0.001.

Administered doses of MZ among all patients were on average 7.3 mg (median 8 mg, range 2.7–14 mg). The distribution of doses in primary and specialist dental clinics differed significantly (*p* < 0.001, Figure 2). The primary dental clinics (mean dose 7.9 mg) used higher doses than the specialist clinics (mean dose 6.8 mg). In specialist care, MZ was combined with inhalation of nitrous oxide on 39 occasions in 16 unique patients (MZ average dose 5.4 mg, median 4 mg, range 4–8 mg). Doses given to patients at different ages are shown in Table 2. Patients older than 70 years received lower doses (mean dose 3.6 mg) compared to patients aged 20–54 years (mean dose 7.5 mg, *p* < 0.001) and 55–70 years (mean dose 7.3 mg, *p* < 0.001), while no difference was observed comparing age groups 20–54 with 55–70 years. Weight was registered on 88 (21.1%) sedation occasions (69 [26.0%] unique patients) and in 85 of these, the dose guidelines had been followed. In three sedation occasions of aged 56–66 years, the dose deviated marginally.

**Table 2:** Given doses of MZ in different age groups.

Variable	20–54 years	55–70 years	71 years –

	*n* = 319	*n* = 86	*n* = 13
Dose mg, average^1^	7.5	7.3	3.6
Dose mg, median	8	8	3
Dose mg, range	4–14	4–10	2.7–6
Age years, mean	34.0	60.0	79.2
ASA 1, number of patients	199	37	2
ASA 2, number of patients	41	16	1
ASA 3, number of patients	2	1	1
ASA 4, number of patients	0	0	0
ASA not described	77	32	9
Weight specified	71	16	1

1 Significant difference 20–54 years versus ≥71 years and 55–70 years versus ≥ 71 years, Linear regression analysis *p* < 0.001.

Dose by age group (side effect) analysed with linear regression analysis. Sedation occasions *n* = 418.

**Figure 3:**
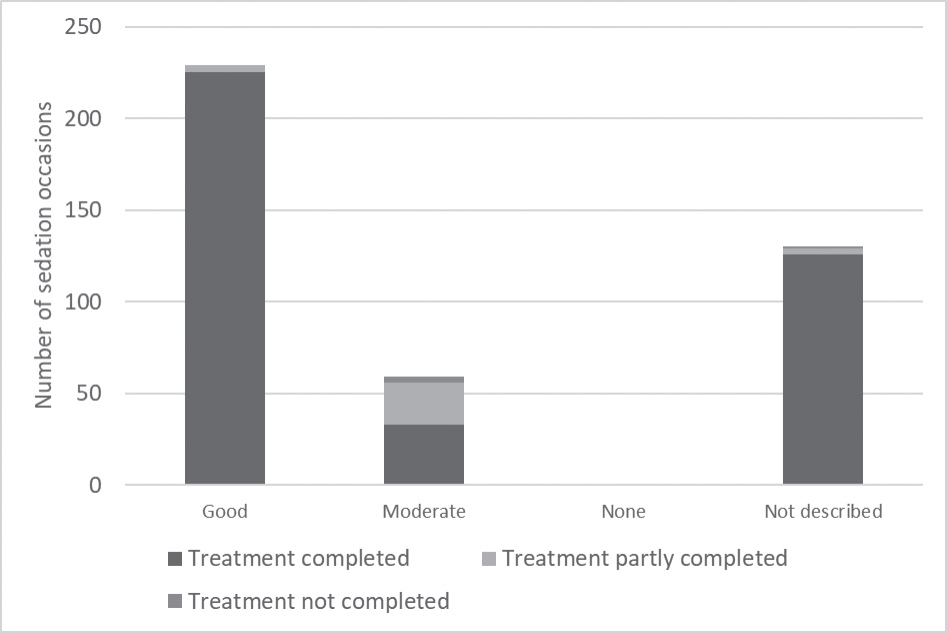
Effect of sedation related to the likelihood of completing treatment. Sedation occasions *n* = 418.

Therapists’ description of the effect of the sedation was translated into the levels good, moderate and none. The rating ‘good’ was chosen for a majority of the sedations (54.8%), ‘moderate’ in 14.1% while ‘none’ was not selected at all. In almost one third of the treatments (*n* = 130), the effect was not described. The treatment could be carried out in 91.9% of the sedation occasions, and in the 130 cases where the effect could not be identified in the medical records, the treatment had been carried out in 96.9% of occasions (Figure 3). We observed no differences in age, dose, or gender distribution, between completed and non-completed treatments (Table 3). However, there was a difference in the type of planned treatment (*p* < 0.001), for example, all 68 tooth extractions were completed compared with nine of the 19 depurations. In addition, fewer patients in specialist dental care compared with primary clinics completed the treatment (*p* < 0.001, Table 3). On 97 occasions, the time between intake of MZ and complete sedation was given. The average time was 22 min (median 20 min, range 5–38 min).

**Table 3:** Distribution of gender, age, planned treatment, dose and character of clinic in patients who completed versus did not complete treatment.

Description	Not completed	Completed	*P*

	*n* = 22	*n* = 243	
**Gender**			
Female	12 (54.5)	171 (70.4)	0.149
Male	10 (45.5)	72 (29.6)	
**Age (years)**			
Continuous	38.1 (14.5)	39.9 (14.3)	0.577
**Planned treatment**			< 0.001
Depuration	10 (45.5)	9 (3.7)	
Emergency visits	1 (4.5)	13 (5.3)	
Filling	6 (27.3)	48 (19.8)	
Others	1 (4.5)	7 (2.9)	
Root canal treatment	3 (13.6)	33 (13.6)	
Surgical operation	1 (4.5)	64 (26.3)	
Tooth extraction		68 (28.0)	
Missing		1 (0.4)	
**Dose (mg) Continuous**	7.7 (2.1)	7.5 (1.3)	0.561
**Clinical character**			
Primary dental clinic	4 (18.2)	147 (60.5)	< 0.001
Specialist clinic	18 (81.8)	96 (39.5)	

Patients first visit during the year studied. Continuous variables analyzed with two-sample t-test, category variables with Fischer’s exact test. Mean and SD or *n* (%). Unique patients *n* = 265.

The records were searched to find factors that increase security during sedation. Due to lack of standardization in the record notes, data were often missing (Table 1). A medical history was found in 98.1% of the records and was the most comprehensive information in the medical records. ASA classification was shown for 71.8% of the sedations, while 118 sedation occasions lacked this information (Table 1). Fasting before sedation and use of pulse oximetry were described in half of the sedations, respectively. Relative contraindications were reported for 14 unique patients who received sedation on a total of 23 occasions. Examples of contraindications were asthma and use of several psychotropic drugs (data not shown). The average dose MZ was 6.4 mg in ages 20–54 years and 5.9 mg for the four patients who were aged 55–70 years. The treatment could be carried out in 22 of the 23 occasions (95.7%), a proportion that is equal or even better than the whole sample. The majority (66.6%) of the sedations on patients with a noted contraindication were performed at a specialist clinic. On nine occasions unfavorable side effects were reported. Dose of MZ was slightly lower (mean 7.0 mg, range 4–8 mg) in sedation occasions where there were side effects compared to sessions without side effects, and in three cases, MZ was combined with nitrous oxide. In five sedation occasions, the patients became nauseous, in three cases drowsier than expected, and in one the patient became hyperactive. A medical history was available in all cases except one, and no contraindications were reported. In all cases, the planned treatment could be carried out despite the side effects, indicating that the incidents were not serious. Males more often experienced side effects than females (*p* < 0.026), but no differences could be noted regarding age, reason for indication, and type of treatment. In no cases were any special measures required to treat the side effect. In 78 sedations, the absence of side effects was documented, but on 331 occasions, side effects were not mentioned at all. In the statistical analysis, it was assumed that if side effects were not mentioned, no side effects had occurred.

## Discussion

This study aimed to map the use of orally administered MZ in a dental care organization. A total of 179,784 dental care visits were carried out in the organization during the year studied, and in 418 of these visits, MZ was used. A retrospective record review including all adult patients who received MZ was performed. Data about patient characteristics and achieved sedative effects showed that MZ administered by dentists seems to be safe and effective.

People 55 years and older were more rarely offered sedation although a previous study found MZ sedation effective and safe even at older ages [2]. This may be explained by many older people having access to sedative drugs through other clinicians, for example, doctors in nursing homes. Another explanation may be that the prevalence of dental anxiety seems to decrease with increased age [19]. However, a repeated cross-sectional study reported that dental anxiety had decreased only among 10–15-year-olds but remained unchanged at older ages [20]. Other studies suggest several reasons for avoiding sedation, such as lack of training among clinicians, increased cost for both clinicians and patients, and patients’ fears of sedation [8].

Two-thirds of the sedated patients were females, which is adequate as dental anxiety in Swedish populations has been reported to be more common among females [3]. According to Svensson et al. [3], 19% of the population suffer from dental anxiety to varying degrees. Thus, dental anxiety still remains a clinical reality with the potential to cause problems for both patients and clinicians. In this study, MZ was used in 0.33% of treated patients, a very low proportion compared to the prevalence of dental anxiety in the population, indicating that more patients in this study may have benefited from sedation. Coulthard [6] estimates that 6.7% of the population would benefit from conscious sedation, with an even higher need for more invasive treatments. In the present study, the use of MZ was higher in specialist dentistry than in primary dental care. This may be explained by the more complicated patient and treatment profiles in specialist dental care.

The guidelines in dentistry for MZ doses were 0.2 mg/kg body weight for adults <55 years, 0.1 mg/kg 55–70 years, and 0.05 mg/kg >70 years. Administered doses of MZ among all patients in this study were on average 7.3 mg with no differences between ages 20 and 54 compared to ages 55–70 years despite the guidelines. Patients aged 71 years and older received lower doses in accordance with the guidelines, but weight was specified for only 18% of the patients, even though weight is necessary to calculate a correct dose. One reason why weight was not specified may be that the maximum dose in guidelines is already reached at 40 kg body weight for people under 55 years of age, which means that for all patients >40 kg, the maximum dose will not change regardless of patients’ actual weight. However, knowledge of the patient’s body weight is essential for safe care, especially for patients older than 55 years where the maximum dose is easily exceeded if the weight is not known. In the study by Rignell et al. [2] where patients aged 62–93 years were sedated, the average body weight was 61.5 kg, and the average MZ dose given was 0.11 mg/kg. Although weight was not recorded for most patients, in most sedation occasions, the therapists seem to have chosen doses that were within the limits of the guidelines. This is also supported by the compliance to the guidelines when the weight was described. While primary dentistry gave the maximum dose in 91% of sedation occasions, specialist dentistry more often adjusted the dose. Reasons for this could be that specialist dentistry had older and probably also sicker patients. In addition, specialist dental care had the opportunity to supplement the MZ sedation with nitrous oxide. Nevertheless, there were more patients in specialist dental care who were unable to complete planned treatment compared to primary dentistry.

The effect of the sedation was registered as good, moderate, or none, but the effect was not described in one-third of the treatments. As the dental treatments could be carried out in 92% of occasions, and in cases where the effect was not documented, even 97%, it was assumed that the effect measured by completed clinical procedures was sufficient. Hence, the impact of patient and procedural characteristics on the effect of sedation was assessed based on the risk of not being able to complete the treatment instead of the therapists’ assessment in terms of good, moderate, or none. In a Swedish study of 61 patients aged 62–93 years sedated with oral MZ, 87% showed no or minor cooperation problems while it was not possible to treat three individuals [2]. In an RCT-study performed in Norway, dental treatments supported by MZ sedation were shown to reduce dental anxiety significantly [21]. Heard et al. [12] reported successful dental treatments in 83% of children with mean age 3.9 years who were sedated with oral MZ. Li et al. [22] found a success rate of oral MZ during tooth extraction of 75.6% in a meta-analysis of a mainly pediatric population. In a systematic review, in which four trials with 359 adults and 77 children compared oral MZ with placebo, MZ reduced ratings of pain and anxiety, but it was unclear whether MZ had an effect on performing clinical procedures [11, 13]. The result of the present study seems to be concordant with the previous studies. However, an unexpected result in the study was that the dental treatment that failed most often was depuration, while tooth extraction was successful most frequently. The 24 individuals who required MZ during depuration differed in terms of reason for sedation as 71% had a disability compared to 14% for the entire sample. In patients with intellectual disability poor ability to cooperate with dental treatments is common, and the risk of impaired oral health is imminent [23]. For these groups, a combination of several techniques, for example, MZ and nitrous oxide, may be necessary to obtain sufficient sedation effects [22, 24].

Several studies report few complications and side effects related to oral MZ sedation regardless of which patient group was studied [2, 12, 15, 16]. However, standardized terminology and reporting of side effects are largely lacking, which make comparisons difficult [16]. A systematic review by Araujo et al. [25], where adults received oral sedation during dental surgical procedures, illustrates the problem with unclear terminology. The frequency of adverse effects described by the patients was reported to be 50–56%, but the adverse events are described as drowsiness, dizziness, muscular relaxation, and amnesia, that is, effects to be desired and expected from MZ. However, reported side effects in studies are not life threatening or even require special treatment [22]. Orally administered MZ was considered safe in dental treatment of uncooperative older people with major neurocognitive disorder. Rignell et al. [2] considered MZ sedation safe with only two of 61 patients suffering from milder side effects: one became hyperactive and one drowsier than expected. In a study of a pediatric population that compared four sedation techniques, no side effects were detected [12]. In the present study, nine patients, 2.2%, were reported to have experienced side effects. No correlations between side effects and age, indication for sedation, or type of treatment, could be noted in the analysis but still cannot be excluded as patients with complications were too few to draw conclusions. None of the side effects required special treatments and can therefore be considered unusual and mild. However, as sedation template was not frequently used in this study, important data for patient safety such as weight and ASA classification were often missing.

This study has several strengths and limitations. Strengths are that the study is a total survey of all dental sedation occasions performed in public dental health in a county in Sweden, apart from one clinic that could not deliver data. This means that 95% of the dental visits could be included. The observations were made over a limited period of one year, which means that the same conditions, such as guidelines and drug composition, were applied throughout the entire period. Another strength is the large number of observations. However, the retrospective design of the study might result in an inherent bias and limitations. Prospective studies can create study groups with similar characteristics and use standardized protocols. Retrospective study data are often unstructured or missing without the possibility of follow-up. Although recommended in the guidelines, only 18% of the sedation occasions in this study used a separate sedation record. Background data, such as medical history, ASA classification, contraindications, and disabilities, could often be retrieved from elsewhere in the medical record, but clinical data about the current sedation were too often missing. This may have resulted in missing or incomplete data that meant that some analyses could not be performed. In addition, a large number of dentists performed the sedations, which presents further risks of the data becoming compromised. On the other hand, a retrospective study can provide a better picture of the treatment outcomes in clinical practice. Despite the shortcomings of the study design, the results of the study regarding effect and side effects of MZ are consistent with previous published studies [2, 15, 22, 26, 27].

Anxiety and fear during dental visits are common in the population, as well as cognitive and intellectual impairment that contribute to lack of cooperation in dental care situations. As three out of four patients who report severe dental anxiety still attend dental care regularly [3], the probability is high that primary dental care will treat these patients. Competence and readiness to offer sedation with MZ in first-line dental care can prevent and reduce dental fear and facilitate dental treatment for both patients and practitioners. Sedation with MZ can also facilitate dental treatment for people with intellectual disabilities or dementia and thereby reduce the number of patients who need to be referred to hospital for general anesthesia [1, 28]. To maintain good safety, patients with complex diagnoses can be treated in specialist dentistry. The option to sedate is rarely used, particularly in primary dental care, and the use varies widely between clinics. However, sedation with MZ administered by dentists both in primary dental care and specialist care seems to be safe and effective.
